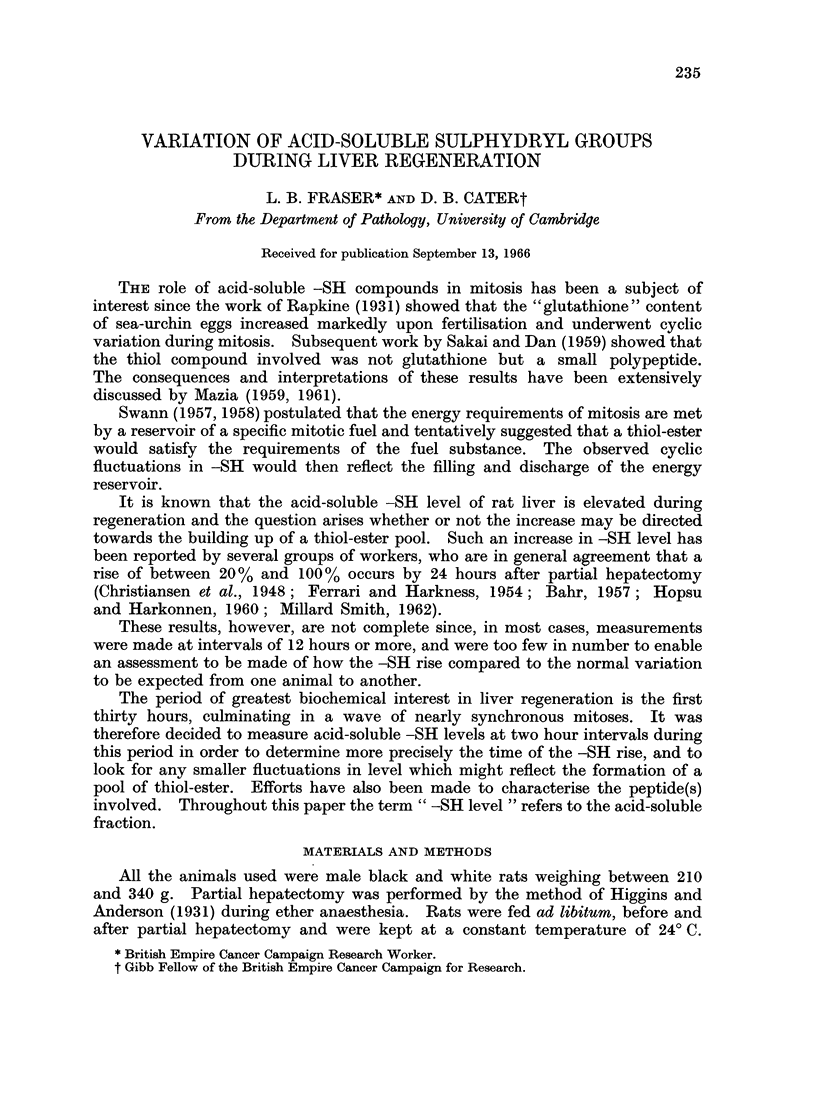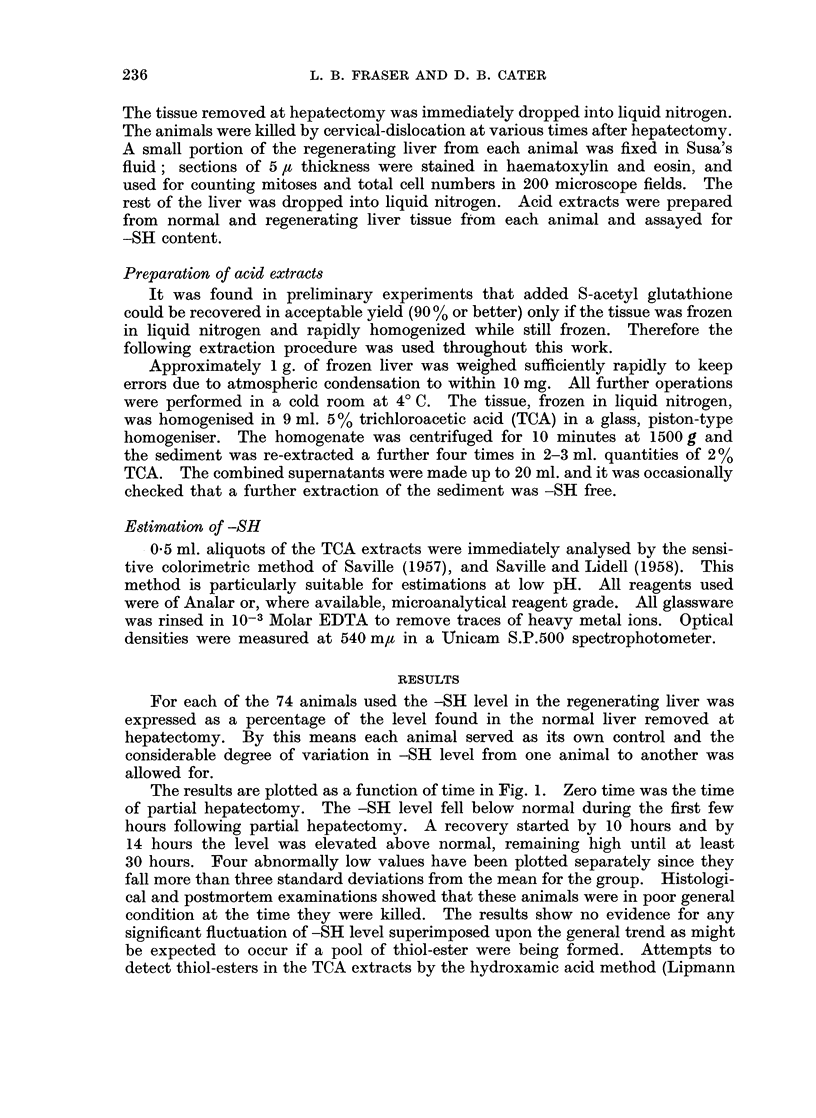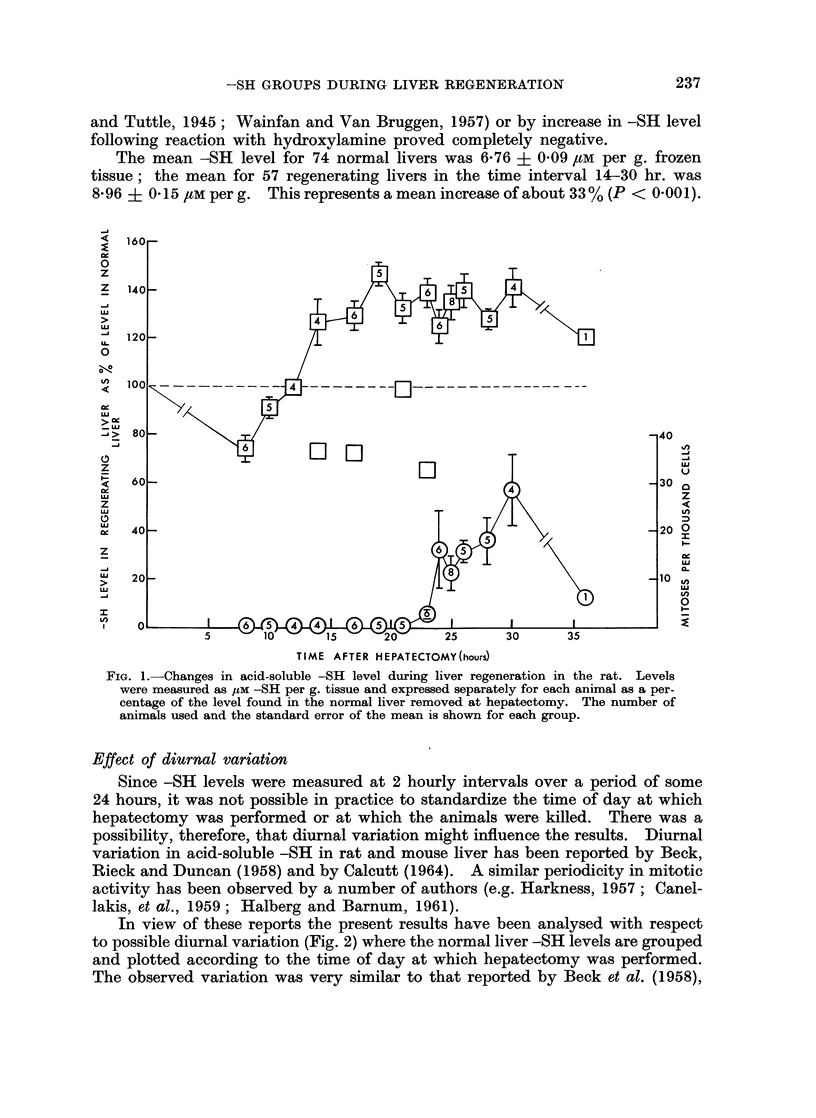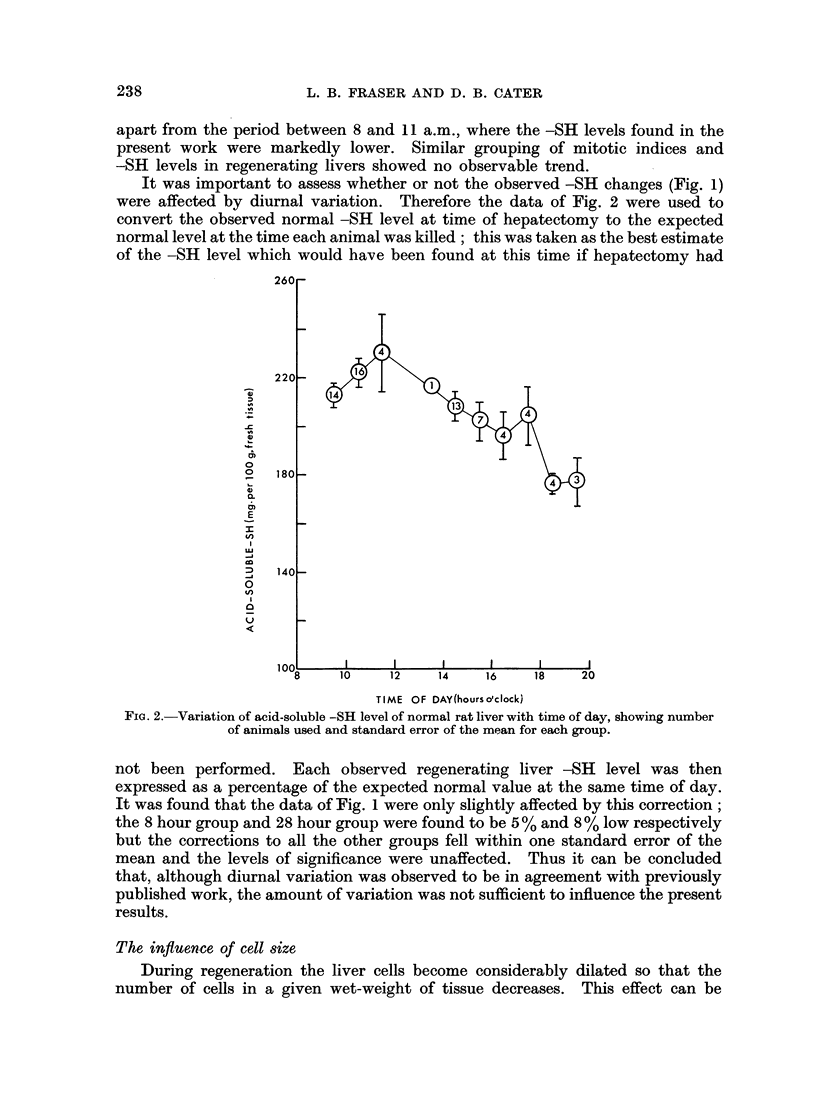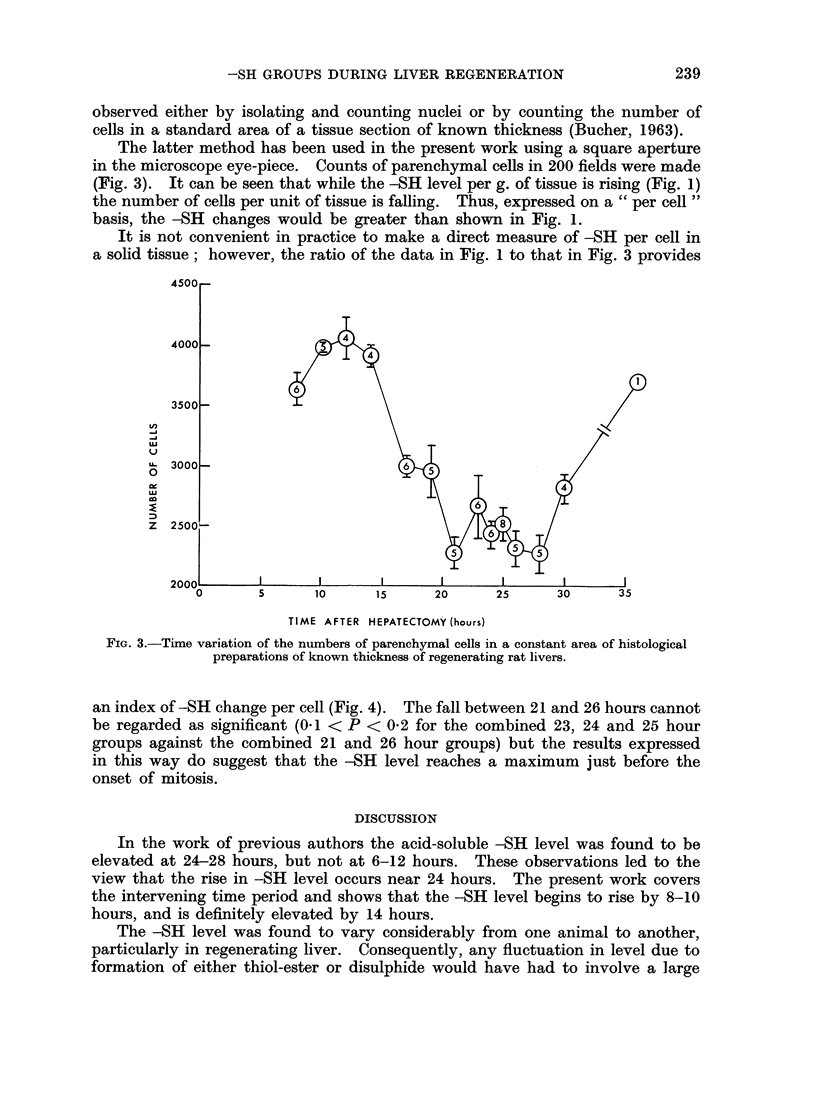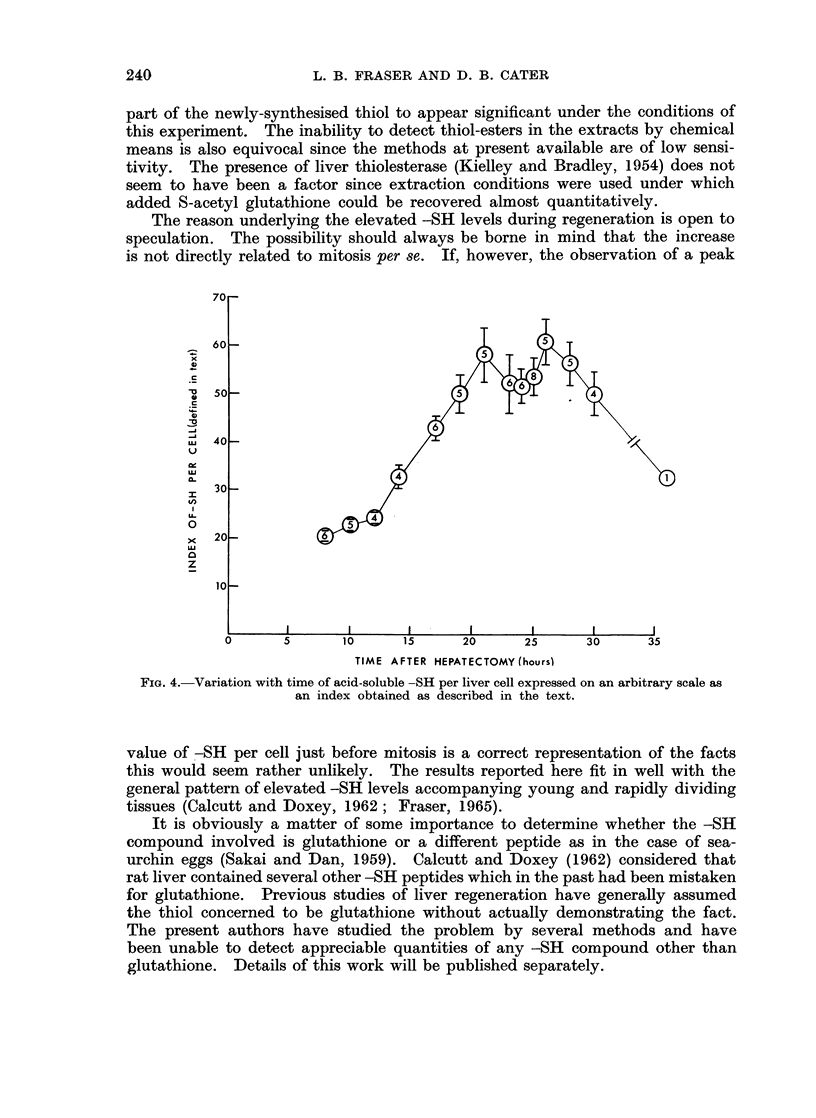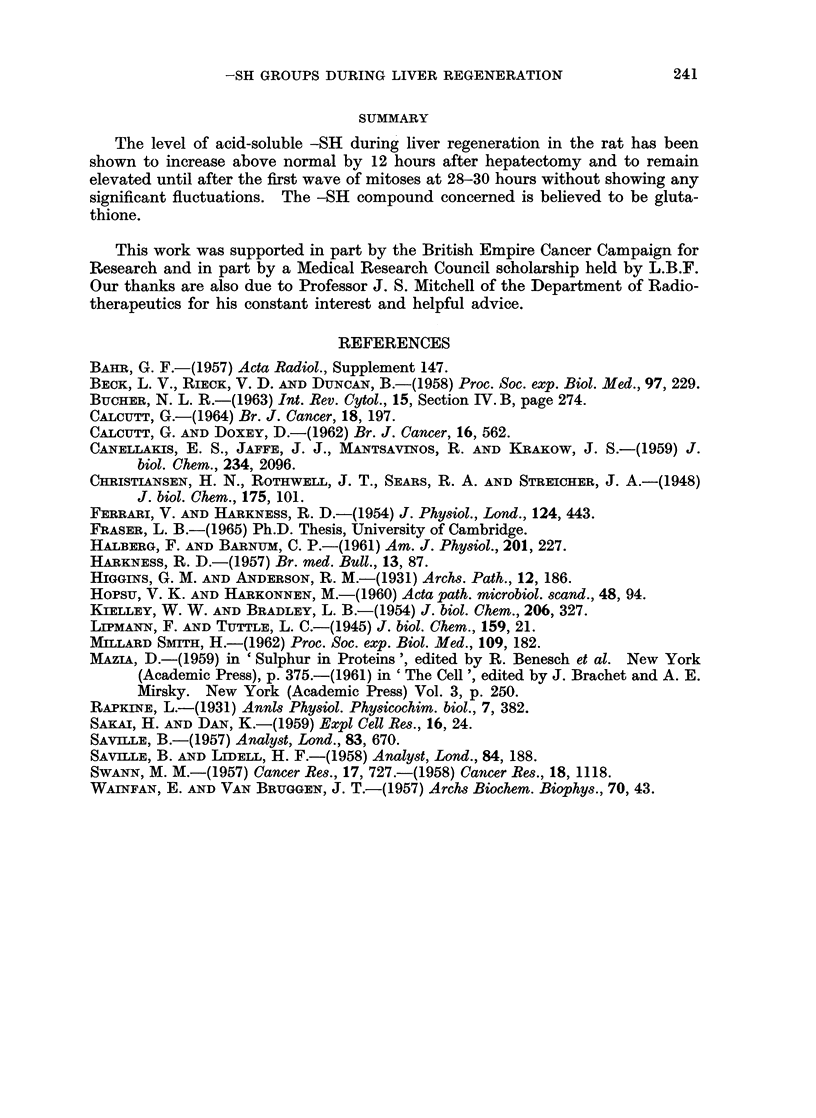# Variation of acid-soluble sulphydryl groups during liver regeneration.

**DOI:** 10.1038/bjc.1967.26

**Published:** 1967-03

**Authors:** L. B. Fraser, D. B. Cater


### VARIATION OF ACID-SOLUBLE SULPHYDRYL GROUPS DURING LIVER REGENERATION

L. B. FRASER* AND D. B. CATER†
*From the Department of Pathology, University of Cambridge*

Received for publication September 13, 1966

THE role of acid-soluble –SH compounds in mitosis has been a subject of interest since the work of Rapkine (1931) showed that the "glutathione" content of sea-urchin eggs increased markedly upon fertilisation and underwent cyclic variation during mitosis. Subsequent work by Sakai and Dan (1959) showed that the thiol compound involved was not glutathione but a small polypeptide. The consequences and interpretations of these results have been extensively discussed by Mazia (1959, 1961).

Swann (1957, 1958) postulated that the energy requirements of mitosis are met by a reservoir of a specific mitotic fuel and tentatively suggested that a thiol-ester would satisfy the requirements of the fuel substance. The observed cyclic fluctuations in –SH would then reflect the filling and discharge of the energy reservoir.

It is known that the acid-soluble –SH level of rat liver is elevated during regeneration and the question arises whether or not the increase may be directed towards the building up of a thiol-ester pool. Such an increase in –SH level has been reported by several groups of workers, who are in general agreement that a rise of between 20% and 100% occurs by 24 hours after partial hepatectomy (Christiansen *et al.*, 1948; Ferrari and Harkness, 1954; Bahr, 1957; Hopsu and Harkonnen, 1960; Millard Smith, 1962).

These results, however, are not complete since, in most cases, measurements were made at intervals of 12 hours or more, and were too few in number to enable an assessment to be made of how the –SH rise compared to the normal variation to be expected from one animal to another.

The period of greatest biochemical interest in liver regeneration is the first thirty hours, culminating in a wave of nearly synchronous mitoses. It was therefore decided to measure acid-soluble –SH levels at two hour intervals during this period in order to determine more precisely the time of the –SH rise, and to look for any smaller fluctuations in level which might reflect the formation of a pool of thiol-ester. Efforts have also been made to characterise the peptide(s) involved. Throughout this paper the term " –SH level " refers to the acid-soluble fraction.

### MATERIALS AND METHODS

All the animals used were male black and white rats weighing between 210 and 340 g. Partial hepatectomy was performed by the method of Higgins and Anderson (1931) during ether anaesthesia. Rats were fed *ad libitum*, before and after partial hepatectomy and were kept at a constant temperature of 24° C.

* British Empire Cancer Campaign Research Worker.
† Gibb Fellow of the British Empire Cancer Campaign for Research.

The tissue removed at hepatectomy was immediately dropped into liquid nitrogen. The animals were killed by cervical-dislocation at various times after hepatectomy. A small portion of the regenerating liver from each animal was fixed in Susa's fluid; sections of 5 $\mu$ thickness were stained in haematoxylin and eosin, and used for counting mitoses and total cell numbers in 200 microscope fields. The rest of the liver was dropped into liquid nitrogen. Acid extracts were prepared from normal and regenerating liver tissue from each animal and assayed for –SH content.

*Preparation of acid extracts*

It was found in preliminary experiments that added S-acetyl glutathione could be recovered in acceptable yield (90% or better) only if the tissue was frozen in liquid nitrogen and rapidly homogenized while still frozen. Therefore the following extraction procedure was used throughout this work.

Approximately 1 g. of frozen liver was weighed sufficiently rapidly to keep errors due to atmospheric condensation to within 10 mg. All further operations were performed in a cold room at 4° C. The tissue, frozen in liquid nitrogen, was homogenised in 9 ml. 5% trichloroacetic acid (TCA) in a glass, piston-type homogeniser. The homogenate was centrifuged for 10 minutes at 1500 *g* and the sediment was re-extracted a further four times in 2–3 ml. quantities of 2% TCA. The combined supernatants were made up to 20 ml. and it was occasionally checked that a further extraction of the sediment was –SH free.

*Estimation of –SH*

0·5 ml. aliquots of the TCA extracts were immediately analysed by the sensitive colorimetric method of Saville (1957), and Saville and Lidell (1958). This method is particularly suitable for estimations at low pH. All reagents used were of Analar or, where available, microanalytical reagent grade. All glassware was rinsed in $10^{-3}$ Molar EDTA to remove traces of heavy metal ions. Optical densities were measured at 540 m$\mu$ in a Unicam S.P.500 spectrophotometer.

### RESULTS

For each of the 74 animals used the –SH level in the regenerating liver was expressed as a percentage of the level found in the normal liver removed at hepatectomy. By this means each animal served as its own control and the considerable degree of variation in –SH level from one animal to another was allowed for.

The results are plotted as a function of time in Fig. 1. Zero time was the time of partial hepatectomy. The –SH level fell below normal during the first few hours following partial hepatectomy. A recovery started by 10 hours and by 14 hours the level was elevated above normal, remaining high until at least 30 hours. Four abnormally low values have been plotted separately since they fall more than three standard deviations from the mean for the group. Histological and postmortem examinations showed that these animals were in poor general condition at the time they were killed. The results show no evidence for any significant fluctuation of –SH level superimposed upon the general trend as might be expected to occur if a pool of thiol-ester were being formed. Attempts to detect thiol-esters in the TCA extracts by the hydroxamic acid method (Lipmann and Tuttle, 1945 ; Wainfan and Van Bruggen, 1957) or by increase in –SH level following reaction with hydroxylamine proved completely negative.

The mean –SH level for 74 normal livers was 6·76 ± 0·09 μM per g. frozen tissue ; the mean for 57 regenerating livers in the time interval 14–30 hr. was 8·96 ± 0·15 μM per g. This represents a mean increase of about 33% ($P < 0{\cdot}001$).

FIG. 1.—Changes in acid-soluble –SH level during liver regeneration in the rat. Levels were measured as μM –SH per g. tissue and expressed separately for each animal as a percentage of the level found in the normal liver removed at hepatectomy. The number of animals used and the standard error of the mean is shown for each group.

*Effect of diurnal variation*

Since –SH levels were measured at 2 hourly intervals over a period of some 24 hours, it was not possible in practice to standardize the time of day at which hepatectomy was performed or at which the animals were killed. There was a possibility, therefore, that diurnal variation might influence the results. Diurnal variation in acid-soluble –SH in rat and mouse liver has been reported by Beck, Rieck and Duncan (1958) and by Calcutt (1964). A similar periodicity in mitotic activity has been observed by a number of authors (e.g. Harkness, 1957 ; Canellakis, *et al.*, 1959 ; Halberg and Barnum, 1961).

In view of these reports the present results have been analysed with respect to possible diurnal variation (Fig. 2) where the normal liver –SH levels are grouped and plotted according to the time of day at which hepatectomy was performed. The observed variation was very similar to that reported by Beck *et al.* (1958), apart from the period between 8 and 11 a.m., where the –SH levels found in the present work were markedly lower. Similar grouping of mitotic indices and –SH levels in regenerating livers showed no observable trend.

It was important to assess whether or not the observed –SH changes (Fig. 1) were affected by diurnal variation. Therefore the data of Fig. 2 were used to convert the observed normal –SH level at time of hepatectomy to the expected normal level at the time each animal was killed ; this was taken as the best estimate of the –SH level which would have been found at this time if hepatectomy had

FIG. 2.—Variation of acid-soluble –SH level of normal rat liver with time of day, showing number of animals used and standard error of the mean for each group.

not been performed. Each observed regenerating liver –SH level was then expressed as a percentage of the expected normal value at the same time of day. It was found that the data of Fig. 1 were only slightly affected by this correction ; the 8 hour group and 28 hour group were found to be 5% and 8% low respectively but the corrections to all the other groups fell within one standard error of the mean and the levels of significance were unaffected. Thus it can be concluded that, although diurnal variation was observed to be in agreement with previously published work, the amount of variation was not sufficient to influence the present results.

*The influence of cell size*

During regeneration the liver cells become considerably dilated so that the number of cells in a given wet-weight of tissue decreases. This effect can be observed either by isolating and counting nuclei or by counting the number of cells in a standard area of a tissue section of known thickness (Bucher, 1963).

The latter method has been used in the present work using a square aperture in the microscope eye-piece. Counts of parenchymal cells in 200 fields were made (Fig. 3). It can be seen that while the –SH level per g. of tissue is rising (Fig. 1) the number of cells per unit of tissue is falling. Thus, expressed on a " per cell " basis, the –SH changes would be greater than shown in Fig. 1.

It is not convenient in practice to make a direct measure of –SH per cell in a solid tissue ; however, the ratio of the data in Fig. 1 to that in Fig. 3 provides

FIG. 3.—Time variation of the numbers of parenchymal cells in a constant area of histological preparations of known thickness of regenerating rat livers.

an index of –SH change per cell (Fig. 4). The fall between 21 and 26 hours cannot be regarded as significant ($0{\cdot}1 < P < 0{\cdot}2$ for the combined 23, 24 and 25 hour groups against the combined 21 and 26 hour groups) but the results expressed in this way do suggest that the –SH level reaches a maximum just before the onset of mitosis.

### DISCUSSION

In the work of previous authors the acid-soluble –SH level was found to be elevated at 24–28 hours, but not at 6–12 hours. These observations led to the view that the rise in –SH level occurs near 24 hours. The present work covers the intervening time period and shows that the –SH level begins to rise by 8–10 hours, and is definitely elevated by 14 hours.

The –SH level was found to vary considerably from one animal to another, particularly in regenerating liver. Consequently, any fluctuation in level due to formation of either thiol-ester or disulphide would have had to involve a large part of the newly-synthesised thiol to appear significant under the conditions of this experiment. The inability to detect thiol-esters in the extracts by chemical means is also equivocal since the methods at present available are of low sensitivity. The presence of liver thiolesterase (Kielley and Bradley, 1954) does not seem to have been a factor since extraction conditions were used under which added S-acetyl glutathione could be recovered almost quantitatively.

The reason underlying the elevated –SH levels during regeneration is open to speculation. The possibility should always be borne in mind that the increase is not directly related to mitosis *per se*. If, however, the observation of a peak

Fig. 4.—Variation with time of acid-soluble –SH per liver cell expressed on an arbitrary scale as an index obtained as described in the text.

value of –SH per cell just before mitosis is a correct representation of the facts this would seem rather unlikely. The results reported here fit in well with the general pattern of elevated –SH levels accompanying young and rapidly dividing tissues (Calcutt and Doxey, 1962 ; Fraser, 1965).

It is obviously a matter of some importance to determine whether the –SH compound involved is glutathione or a different peptide as in the case of sea-urchin eggs (Sakai and Dan, 1959). Calcutt and Doxey (1962) considered that rat liver contained several other –SH peptides which in the past had been mistaken for glutathione. Previous studies of liver regeneration have generally assumed the thiol concerned to be glutathione without actually demonstrating the fact. The present authors have studied the problem by several methods and have been unable to detect appreciable quantities of any –SH compound other than glutathione. Details of this work will be published separately.

SUMMARY

The level of acid-soluble –SH during liver regeneration in the rat has been shown to increase above normal by 12 hours after hepatectomy and to remain elevated until after the first wave of mitoses at 28–30 hours without showing any significant fluctuations. The –SH compound concerned is believed to be glutathione.

This work was supported in part by the British Empire Cancer Campaign for Research and in part by a Medical Research Council scholarship held by L.B.F. Our thanks are also due to Professor J. S. Mitchell of the Department of Radiotherapeutics for his constant interest and helpful advice.

### REFERENCES

Bahr, G. F.—(1957) *Acta Radiol.*, Supplement 147.
Beck, L. V., Rieck, V. D. and Duncan, B.—(1958) *Proc. Soc. exp. Biol. Med.*, **97**, 229.
Bucher, N. L. R.—(1963) *Int. Rev. Cytol.*, **15**, Section IV. B, page 274.
Calcutt, G.—(1964) *Br. J. Cancer*, **18**, 197.
Calcutt, G. and Doxey, D.—(1962) *Br. J. Cancer*, **16**, 562.
Canellakis, E. S., Jaffe, J. J., Mantsavinos, R. and Krakow, J. S.—(1959) *J. biol. Chem.*, **234**, 2096.
Christiansen, H. N., Rothwell, J. T., Sears, R. A. and Streicher, J. A.—(1948) *J. biol. Chem.*, **175**, 101.
Ferrari, V. and Harkness, R. D.—(1954) *J. Physiol., Lond.*, **124**, 443.
Fraser, L. B.—(1965) Ph.D. Thesis, University of Cambridge.
Halberg, F. and Barnum, C. P.—(1961) *Am. J. Physiol.*, **201**, 227.
Harkness, R. D.—(1957) *Br. med. Bull.*, **13**, 87.
Higgins, G. M. and Anderson, R. M.—(1931) *Archs. Path.*, **12**, 186.
Hopsu, V. K. and Harkonnen, M.—(1960) *Acta path. microbiol. scand.*, **48**, 94.
Kielley, W. W. and Bradley, L. B.—(1954) *J. biol. Chem.*, **206**, 327.
Lipmann, F. and Tuttle, L. C.—(1945) *J. biol. Chem.*, **159**, 21.
Millard Smith, H.—(1962) *Proc. Soc. exp. Biol. Med.*, **109**, 182.
Mazia, D.—(1959) in ‘ Sulphur in Proteins ’, edited by R. Benesch *et al.* New York (Academic Press), p. 375.—(1961) in ‘ The Cell ’, edited by J. Brachet and A. E. Mirsky. New York (Academic Press) Vol. 3, p. 250.
Rapkine, L.—(1931) *Annls Physiol. Physicochim. biol.*, **7**, 382.
Sakai, H. and Dan, K.—(1959) *Expl Cell Res.*, **16**, 24.
Saville, B.—(1957) *Analyst, Lond.*, **83**, 670.
Saville, B. and Lidell, H. F.—(1958) *Analyst, Lond.*, **84**, 188.
Swann, M. M.—(1957) *Cancer Res.*, **17**, 727.—(1958) *Cancer Res.*, **18**, 1118.
Wainfan, E. and Van Bruggen, J. T.—(1957) *Archs Biochem. Biophys.*, **70**, 43.